# Role of microRNAs in antiviral responses to dengue infection

**DOI:** 10.1186/s12929-019-0614-x

**Published:** 2020-01-03

**Authors:** Rui Rui Wong, Noraini Abd-Aziz, Sarah Affendi, Chit Laa Poh

**Affiliations:** Centre for Virus and Vaccine Research (CVVR), Sunway University, 47500 Subang Jaya, Selangor Malaysia

**Keywords:** microRNA, Dengue virus, Host-pathogen interactions, Virus replication, Antiviral immunity

## Abstract

Dengue virus (DENV) is the etiological agent of dengue fever. Severe dengue could be fatal and there is currently no effective antiviral agent or vaccine. The only licensed vaccine, Dengvaxia, has low efficacy against serotypes 1 and 2. Cellular miRNAs are post-transcriptional regulators that could play a role in direct regulation of viral genes. Host miRNA expressions could either promote or repress viral replications. Induction of some cellular miRNAs could help the virus to evade the host immune response by suppressing the IFN-α/β signaling pathway while others could upregulate IFN-α/β production and inhibit the viral infection. Understanding miRNA expressions and functions during dengue infections would provide insights into the development of miRNA-based therapeutics which could be strategized to act either as miRNA antagonists or miRNA mimics. The known mechanisms of how miRNAs impact DENV replication are diverse. They could suppress DENV multiplication by directly binding to the viral genome, resulting in translational repression. Other miRNA actions include modulation of host factors. In addition, miRNAs that could modulate immunopathogenesis are discussed. Major hurdles lie in the development of chemical modifications and delivery systems for in vivo delivery. Nevertheless, advancement in miRNA formulations and delivery systems hold great promise for the therapeutic potential of miRNA-based therapy, as supported by Miravirsen for treatment of Hepatitis C infection which has successfully completed phase II clinical trial.

## Introduction

MicroRNAs (miRNAs) are a class of small non-coding RNAs (18–25 nucleotides) which function as key post-transcriptional regulators of gene expression. By binding to the complementary 3′-untranslated regions (3’UTR) of specific messenger RNAs (mRNAs), miRNAs prevent translational events, either through mRNA degradation or translational repression [1]. At present, more than 2000 human miRNAs are known, implying a powerful regulatory role of miRNAs in diverse aspects of development and physiology [2]. miRNA can be considered as master regulators as they regulate the expressions of about 60% of the protein-coding genes in the human genome. A single mRNA can be targeted by different miRNAs or one miRNA can bind to multiple mRNAs [3]. It is therefore not surprising to observe an aberrant expression profile of miRNAs in various human diseases [4].

Viruses are obligate intracellular parasites that rely highly on host metabolic processes to create specific microenvironments required for viral replication and infectivity. In the event of a viral infection, miRNAs are actively involved in the interplay between viruses and hosts, from viral attachment to disease progression. The presence of microRNA Response Elements (MREs) which are generally located in the 5’UTR and 3’UTR of the viral genome, has supported the underlying mechanism of miRNA-mediated antiviral activities [5, 6]. One of the earlier evidence refers to miR-32 which has been reported to effectively restrict primate foamy virus type I replication by targeting the viral genome [7]. Nevertheless, there was also evidence where miRNA directly binds to the viral genome, resulting in enhanced viral genome stability and translation. This unconventional miRNA-mediated effect has been well exemplified by miR-122, a liver-specific miRNA which contributes to Hepatitis C virus (HCV) liver tropism [5]. miR-122 stabilizes the HCV genome by forming an oligomeric complex with the HCV genome. It was reported to bind to two closely target sites (S1 and S2) in the 5’UTR of HCV genome, thereby protecting the viral genome from nucleolytic degradation [8, 9]. On the basis where sequestration of miR-122 in liver cell lines strongly downregulated HCV replication in liver cells, the miR-122 antagonist Miravirsen has been shown to be an antiviral agent against HCV infection.

Similar to other viruses, miRNA-related research of Dengue virus (DENV) has been growing in recent years. DENV is a mosquito-transmitted, positive sense virus which belongs to the Flavivirus genus of the Flaviviridae family. There are four distinct, but closely related serotypes of the dengue virus, namely DENV-1, DENV-2, DENV-3 and DENV-4. Over the last few decades, dengue has become one of the major public health challenges in the world, especially in tropical and subtropical countries. The World Health Organization (WHO, 2017) reported that the incidence of dengue has increased 30-fold in the recent 50 years [10]. Bhatt and associates estimated that 390 million dengue infections occurred annually, of which 96 million cases were manifested clinically [11]. Another study estimated that the risk of dengue infection will rise and could infect 3.9 billion people in 128 countries across the world [12]. As a disease which is endemic in over 100 countries, dengue has put almost half of the world’s population at risk. In this review, we describe the involvement of miRNAs in the host-pathogen interactions of DENV. Interestingly, miRNAs could act as an antiviral regulator or a proviral factor that favors DENV multiplication.

## Dengue virus: host-pathogen interactions

### DENV entry and replication

DENV has a 10.7-kb capped RNA with 5′ and 3′ UTRs flanking a single open reading frame that encodes three structural proteins (C, capsid; prM/M, pre-membrane/membrane; E, envelope) and seven non-structural proteins (NS1, NS2A, NS2B, NS3, NS4A, NS4B, NS5) [13]. Dengue infection starts with a bite by the DENV-infected Aedes mosquitoes, *Aedes aegypti* or *Aedes albopictus*. Upon DENV inoculation into the skin by a mosquito bite, the virus is taken up by the tissue-resident dendritic cells or the macrophages via clathrin-dependent receptor-mediated endocytosis. DENV attaches to dendritic cell-specific intracellular adhesion molecule-3-grabbing non-integrin (DC-SIGN) on dendritic cells [14]. The mannose receptor on macrophages binds to DENV surface proteins via the lectin activity of the carbohydrate-binding domains of the DENV envelope protein [15].

### Host innate immune response against DENV infection

Upon DENV entry into cells, the viral RNA is uncoated and following replication, the dsRNA can be recognized by the host pattern recognition receptors (PRRs). The PRRs include the Toll-like receptors (TLR3, TLR7 and TLR8) and the host RNA sensors comprising the retinoic acid-inducible gene I protein (RIG-1)-like receptors and melanoma differentiation-associated protein 5 (MDA5) [16]. The cytoplasmic helicases, RIG-1 and MDA5, could detect the presence of the virus through binding to viral RNA. Upon viral recognition, there is an activation of interferon regulatory factors and the NF-κB. These torrent of immunological signals could then trigger the induction of the type I interferons (mainly IFN-α and IFN-β), leading to robust production of inflammatory cytokines including IL-8. Subsequently, dendritic cells are activated with the establishment of an antiviral response [16, 17].

Toll-like receptor 3 (TLR3) was shown to have a pivotal role in the recognition and restriction of DENV replication in several cellular lineages [16, 18, 19]. An in vitro study demonstrated the synergism of RIG-I, MDA5 and TLR3 in the induction of IFN-β production that eventually suppressed DENV replication [18]. Binding of RIG-1 and MDA5 to viral RNA activate the phosphorylation of interferon regulatory factor 3 (IRF3), which in turn initiated IFN production [17, 18]. Although type I IFN suppression has been well recognized as an apparent pathogenic strategy of DENV to evade the host immune response, evidence has shown that there was a relatively mild activation of IFN-α and IFN-β during DENV infection [20–22].

To date, a number of DENV proteins have been identified as the inhibitors of IFN-α/β signaling pathway by interfering with different targets along the pathway. DENV NS2A, NS4A and NS4B have been shown to work in concert to block the phosphorylation of the signal transducer and activator of transcription 1 (STAT1) and nuclear translocation [23]. Another DENV protein, NS5, has been shown to bind directly to signal transducer and activator of transcription 2 (STAT2) protein, which subsequently directed it towards proteasomal degradation [24]. As a result, the formation of STAT1/STAT2 heterodimer and its subsequent transcriptional induction of interferon stimulated genes (ISGs) was prevented. In addition, recent studies have discovered a novel immune evasion strategy of DENV. The viral protease NS2B3 enzymatically cleaved the human mediator of interferon regulatory factor 3 (IRF3) activation (MITA), also known as the stimulator of interferon genes (STING), which downregulated IFN production [25, 26]. The other study discovered that DENV NS4A was able to bind to the mitochondrial antiviral signaling adaptor (MAVS), thereby preventing it from associating with RIG-1 and abrogating IFN induction [27].

### Immunopathogenesis of DENV infection

Dengue fever is a non-fatal disease with symptoms like high fever, headache, vomiting, pain behind the eyes, muscle and joint pain as well as a characteristic skin rash. Unfortunately, a small but significant proportion of dengue patients could develop the Dengue Haemorrhagic Fever (DHF) and the Dengue Shock Syndrome (DSS) which are life-threatening. The pathogenesis of severe dengue has been attributed to immunopathogenesis which elicited an abrupt “cytokine storm”, leading to vascular leakage, hypotension and shock [28]. The complexity of DENV infection is due to immune responses that can either be protective or pathogenic. Exposure to any of the four DENV serotypes during primary infection might render life-long protection against the same serotype. Increased risk of severe dengue is highly associated with heterologous secondary infection, which is a subsequent DENV infection with a different serotype. Aggravated clinical manifestations of dengue disease from heterologous secondary infection might be attributed to cross-reactive T cells [29] or the “antibody-dependent enhancement” (ADE) [30]. The dominance of cross-reactivity of antibodies or T-cell responses towards the primary infecting DENV serotype over the secondary infecting serotype was addressed in the “original antigenic sin” concept of secondary DENV infections [29]. The involvement of cross-reactive T cell responses observed in severe dengue manifestations infers the pathogenic role of T cells in DENV infection [31, 32]. The original antigenic sin proposed that cross-reactive memory T cells from primary infection might have a lower avidity against the secondary infecting serotype, which in turn, could result in suboptimal TCR triggering and subsequent production of high levels of TNF-α that contributed to vascular permeability and plasma leakage [33]. Several studies demonstrated the role of CD4+ and CD8+ T cells in the resolution of DENV infection, whereby serotype-specific responses of the T cells were seen in primary infection of DENV in humans [34], and proliferation of CD4+ T cells responding specifically the NS3 produced IFN-γ that lysed infected cells [35]. Nevertheless, skewed T-cell responses during secondary heterologous infection might contribute to the severity of the immunopathogenesis of DHF and/or DSS. Although secondary DENV infections are either asymptomatic or display a mild outcome of the disease, the immune mechanisms underlying disease severity in some individuals during heterologous infections warrant further research.

## miRNA biogenesis

The biogenesis of miRNAs in humans involves 4 key enzymes- Drosha, Exportin 5, Dicer and Argonaute (Ago) 2 [1, 36]. The process commenced with the transcription of genes encoding miRNAs in the nucleus by the RNA polymerase II, producing primary transcripts which are several kilobases long, namely the pri-miRNAs [37–39]. In the nucleus, the pri-miRNAs are processed into pre-miRNAs by the nuclear Microprocessor complexes consisting of the Ribonuclease III endonuclease Drosha and the co-factor protein DiGeorge syndrome chromosomal region 8 (DGCR8). The pre-miRNAs are approximately 70 nucleotides long hairpin structures [40–42]. They are subsequently transported by Exportin-5 to the cell cytoplasm [43–45] where the Dicer protein enzymatically cleaves the stem-loop precursor to 22 nucleotides long double-stranded miRNA duplexes which are incorporated into the Ago proteins, Ago1 and Ago2, in the miRNA-induced silencing complexes (miRISC) [46–50]. Following this, the duplexes rapidly unwound, giving rise to the mature miRNA guide strand and the complementary passenger strand, known as the miRNA star (miR*). Whilst the mature miRNA is retained in the miRISC, the miR* is released and degraded in the cytoplasm [51, 52]. miRNA-incorporated RISC complex is ready to mediate post-transcriptional silencing by two different mechanisms, depending on the complementarity with target mRNA sequences. A perfect complementarity match generally leads to mRNA degradation, as a result of the endonuclease activity by Ago2. On the other hand, translational inhibition occurs as the outcome of an imperfect complementary binding of RISC to the target sequences [53].

## miRNA-based therapeutic modalities

In general, there are two miRNA-based therapeutic modalities: miRNA antagonists and miRNA mimics. The former approach could be applied to inhibit miRNAs with undesired functions such as miRNAs which are proviral. A miRNA antagonist is usually introduced as a chemically modified miRNA which is known as anti-miR or antagomiR that could complementarily bind to the mature miRNA strand. To date, several chemical modifications have been introduced to antimiR oligonucleotides with the aim of increasing their binding affinity, stability and in vivo delivery. The most commonly used chemical modifications include locked nucleic acid (LNA), a bicyclic RNA analogue in which the ribose is locked in a C3’-endo conformation, 2’ribose modifications such as 2-O-methyl (2-MO), 2-fluoro (2-F), 2-O-methoxyethyl (2-MOE) and the modification of phosphorothioate (PS) backbone [54]. Among them, LNA was reported to be the one with the highest affinity towards complementary miRNAs. In fact, this chemical modification was adopted by Santaris Pharma A/S and Hoffman-La Roche as the miRNA chemistry for the very first miRNA therapeutic and administered as antimiR oligonucleotide targeting miR-122 for the treatment of HCV infection [55].

miRNA mimics are synthetic RNA duplexes which mimic endogenous miRNAs. They are introduced to replenish or to further enhance the levels of miRNAs which are crucial to control disease progression. These miRNA mimics provided a promising proof-of-concept for miRNA replacement therapy. The tumor suppressor miR-34 mimic (MRX34) is by far the most clinically advanced therapeutic mimic. Although the MRX34 phase I clinical trial was halted in September 2016 due to five cases of immune-related events [55], the question of whether miR-34 mimic could serve as a good strategy for cancer therapeutic remains elusive. Interestingly, miR-34 family was also reported to have an antiviral role by induction of IFN-responsive genes and release of type I interferon, thereby enhancing the host immune response against DENV infection. This preliminary result highlighted the potential to develop miR-34 mimic as an anti-DENV therapeutic [56].

## Impact of miRNAs on DENV infection

Several studies have implied the important roles of miRNAs in viral infections. Mutations in the main catalytic components of the RNA interference (RNAi) pathway led to an increase in replication of DENV [57], vesicular stomatitis virus (VSV) [58], influenza A virus [59] and human immunodeficiency virus [60, 61] in mammalian cells. To date, at least two independent studies have extensively profiled miRNA expressions in the blood of dengue patients, which consistently showed a significant number of miRNAs that were differentially expressed in response to dengue infection. Tambyah et al. [62] showed a profile of 348 differentially-expressed miRNAs in dengue patients, of which 12 represented the cohort that was specifically altered upon acute dengue infection. Interestingly, this study also pinpointed 17 miRNAs that could potentially be utilized to distinguish mild dengue from severe dengue with complications. Expressions of miR-24-1-5p, miR-512-5p and miR-4640-3p were able to distinguish mild dengue from those displaying liver complications while miR-383 was significantly upregulated in mild dengue when compared to those diagnosed with severe dengue and fluid accumulation. In another study by Ouyang et al. (2016), miRNA PCR arrays showed differential expressions of 12 downregulated and 41 upregulated miRNAs in the sera of DENV-1 patients when compared to healthy controls. Amongst these miRNAs, hsa-miR-21-5p, hsa-miR-146a-5p, hsa-miR-590-5p, hsa-miR-188-5p, and hsa-miR-152-3p were identified as promising non-invasive molecular markers for detecting DENV infection. miR-21-5p and miR146a-5p were functionally involved in inflammation and cell proliferation, and they were significantly different from the control groups, indicating their high sensitivity and specificity as indicators of DENV infection. In addition, both of the miRNAs correlated with the number of leukocytes and neutrophils. These findings suggested that some miRNAs could be employed as diagnostic markers for DENV infections [63].

Interestingly, it is apparent that an effective disruption of the host RNAi machinery is one of the pathogenic strategies of viruses to mitigate the host response. Several viruses have been reported to produce viral suppressors of RNAi proteins that could suppress miRNA-mediated silencing of the host RNAi machinery by interfering with RISC loading or by inhibiting the slicing activity of the Ago protein, a component of RISC [64–66]. For DENV, viral RNAi suppressors that could neutralize the host RNAi response were also identified. NS4B was reported to suppress the host RNAi by interfering with the processing of dicer, a key miRNA biogenesis protein [57] whilst NS3 was demonstrated to interfere with the miRNA loading to the Ago1 protein [67].

MicroRNA-like small RNAs have been reported to be produced by RNA viruses such as DENV, West Nile and H5N1 influenza viruses [68–70]. Hussain et al. (2014) showed that the miRNA-like viral small RNA DENV-vsRNA-5 was able to autoregulate DENV replication by targeting the non-structural DENV protein 1 gene and down-regulated DENV replication.

### miRNAs that directly target viral genome to inhibit DENV replication

miRNAs generally induce translational repression by binding to MRE sites of the targeted mRNAs. Consistent with this mode of action, it is not surprising that most of the miRNAs identified to date have been reported to affect DENV replication by targeting the viral genome sequences. Evidence gathered independently pointed to the fact that miRNAs complementarily bind to the 5’UTR or 3’UTR of DENV genome and thereby inhibiting DENV replication. The first evidence that a miRNA could suppress DENV multiplication by directly binding to the viral genome was represented by miR-548 g-3p [71]. In the study, Wen et al. (2015) [71] showed that miR-548 g-3p was able to bind to the Stem Loop A (SLA) promoter in the 5’UTR, which is a key element to DENV RNA synthesis and replication, and downregulated replication of DENV-1 (strain Hawaii), DENV-2 (strain New Guinea C), DENV-3 (strain Philippine H87) and DENV-4 (GZ/ 9809/2012) (Table 1). This inhibitory effect was proposed to arise from the binding of the miR-548 g-3p to the SLA which might physically hinder and thereby, attenuated the interaction between the SLA promoter and NS5, a DENV protein which contains a C-terminal RNA-dependent RNA polymerase domain.

**Table 1:** Examples of miRNAs involved in DENV infection

microRNA	Virus-types	Host system	Effect	Target	Reference
miR-548 g-3p	DENV-1 (strain Hawaii), DENV-2 (strain New Guinea C), DENV-3 (Philippine H87 Strain) and DENV-4 (GZ/ 9809/2012)	U937	Antiviral	Direct: viral 5′ UTR	[71]
miR-133a	DENV-1, DENV-2 (strain New Guinea C) and DENV-4	Vero	Antiviral	Direct: viral 3′ UTR	[72]
miR-484	DENV-1, DENV-2 (strain New Guinea C) and DENV-4	Vero	Antiviral	Direct: viral 3′ UTR	[73]
miR-744	DENV-1, DENV-2 (strain New Guinea C) and DENV-4	Vero	Antiviral	Direct: viral 3′ UTR	[73]
miR-252	DENV-2 (strain New Guinea C)	C6/36	Antiviral	Direct: viral gene E	[74]
Let-7c	DENV-2 (strain New Guinea C) and DENV4 (strain V3361–1956)	Huh7, U937-DC-SIGN	Antiviral	Indirect: BACH1	[75]
miR-30e*	DENV-1 (strain Hawaii), DENV-2 (strain New Guinea C) and DENV-3 (strain H241)	HeLa, U937 and PBMCs	Antiviral	Indirect: IκBα	[76]
miR-34 family (miR-34a, miR-34c, miR-449a and miR-449b)	DENV-2 (strain New Guinea C)	HeLa	Antiviral	Indirect: Wnt pathway	[56]
miR-223	DENV-2 (strain TR1751)	EAhy926 cells	Antiviral	Indirect: STMN1 mRNA	[77]
miR-3614-5p	DENV-2 (strain 16,681)	Primary human macrophage	Antiviral	Indirect: ADAR1 mRNA	[78]
miR-146a	DENV-2 (strain New Guinea C)	Primary human monocytes and THPI cells	Proviral	Indirect: TRAF6	[79]
miR-21	DENV-2 (strain 16,681)	HepG2	Proviral	Direct: NS1 sequence	[80]
mir-150	DENV-2 (strain New Guinea C)	PBMCs from DHF patients	Biomarker for severe disease	Downregulated SOCS1 resulting in lower IFN-γ production	[81]

*Abbreviations*: *DENV* Dengue virus, *UTR* Untranslated region, *BACH1* Basic leucine zipper transcription factor 1, *STMN1* Stathmin 1, *ADAR1* Adenosine deaminase acting on RNA 1, *NS1* Non-structural protein 1, *SOCS1* Suppressor of cytokine signaling, *IFN* Interferon, *TRAF6* Tumor necrosis factor receptor (TNFR)-associated factor 6

Castrillon-Betancur and Urcuqui-Inchima [73] revealed three miRNA candidates which could potentially inhibit DENV replication by means of bioinformatics predictions [72]. They hypothesized that a functional miRNA was conserved amongst all DENV serotypes and identified miRNA candidate target sites present at the 3’UTR of all four dengue serotypes. As a result, they proposed that miR-133a, miR-484 and miR-744 could downregulate DENV replication by targeting the 3’UTR of the DENV RNA genome, specifically, the 3’stem loop which contains elements that are involved in genome circularization and viral viability [72, 73]. Indeed, overexpression of miR-133a, miR-484 and miR-744 in Vero cells had experimentally been validated to show the potencies of these three miRNAs in inhibiting DENV replication.

In another study, miR-252 was found to be highly induced in a DENV-2 (strain New Guinea C) infection model of the mosquito C6/36 cell line and was able to suppress DENV replication by targeting the DENV-2 E gene [74]. The identification of the E protein as the miRNA target is interesting as this protein was known to have an indispensable role for cell attachment and viral entry [82]. As miR-252 is not present in humans, administration of miR-252 mimics to prevent the translation of the E protein in humans might act as an antiviral therapeutic with no or minimal off-target effects. Nonetheless, experiments are required to confirm the importance of miR-252 in the context of DENV-2 human infection.

### miRNAs that modulate host factors to inhibit or facilitate DENV replication

As viruses are dependent on the host machinery for replication and infection, it is of no surprise that a number of miRNAs have been demonstrated to indirectly regulate DENV replication through the modulation of the host factors or immune response. These indirect effects included modulation of expression of a cellular transcript encoding a host factor required for one or a few steps in the viral cycle. Modulation of receptor expression could regulate the entry of the virus, thus affecting tropism and cofactors vital for replication complexes or translation that could impair or increase viral replication as well as viral protein production, respectively. In addition, miRNAs are also known to enhance or restrict cell responses to the infection like immune response or defense mechanisms [83].

For example, the highly expressed let-7c has been speculated to protect the infected Huh-7 cells from oxidative stress and inflammation response following DENV infection [75]. Let-7c is one of the miRNAs that was up-regulated during DENV infection in the hepatic Huh-7 cells and macrophage-monocytic cell line U937-DC-SIGN. Let-7c was shown to directly bind to the Basic Leucine Zipper Transcription Factor-1 (BACH1), a strong repressor of the anti-inflammatory and anti-oxidant protein Heme Oxygenase 1 (HO-1), and downregulated both DENV-2 (strain New Guinea C) and DENV-4 (strain V3361–1956) infections. As inferred by its anti-inflammatory and anti-oxidant role, let-7c is likely able to protect the host from the viral-induced excessive production of reactive oxygen species and inflammatory response, which is detrimental to the host [84–88]. Furthermore, another study further supported the antiviral role of HO-1 against DENV [89]. HO-1 induction was demonstrated to reduce DENV replication in Huh-7 cells and to delay DENV-2 induced disease and lethality in suckling mice. The HO-1-mediated antiviral effect is likely contributed by the restoration of antiviral IFN response together with the inhibition of DENV protease activity by biliverdin, a by-product of HO-1-catalyzed degradation of heme.

Zhu et al. (2014) demonstrated that the highly expressed miR-30e* during dengue infection suppressed DENV replication by enhancing the host antiviral immune response [76]. When unstimulated, NF-κB was retained in the cytoplasm by IκBα, a negative regulator which de-repressed the translocation of NF-κB into the nucleus upon its degradation [90]. During DENV infection, miR-30e* was shown to exert the antiviral effect by directly targeting the 3’UTR of IκBα, resulting in the degradation of IκBα. This led to the subsequent activation of NF-κB pathway, thus promoting the expression of IFN-β and the downstream ISGs such as OAS1, MxA and IFITM1, as demonstrated in both U937 monocytic and HeLa cells [76]. Similar innate modulating role by the miR-34 family (miR-34a, miR-34c, miR-449a and miR-449b) was also reported in response to infections caused by a number of flaviviruses, including DENV [56]. A reduction in DENV-2 (strain New Guinea C) replication was observed upon increased expression of the miR-34 family members where the antiviral effect was proposed to be due to the suppression of the Wnt signaling. Wnt signaling is known to repress glycogen synthase kinase 3 beta (GSK3b) phosphorylation which positively regulated the IFN signaling pathway. As a result, miR-34 family increased the expression of type I IFN and ISGs through suppression of Wnt signaling [56]. Hence, both miR-30e* and miR-34 family of miRNAs could be considered as positive regulators of the antiviral immune response of cells upon DENV infections. The ability of both miRNAs to upregulate the expression of type I IFN production indicated their therapeutic potentials in suppressing DENV infections in vivo.

Apart from modulating the host immune response, a study by Wu et al. (2014) provided evidence to link the antiviral role of miRNA to perturbations of other host factors. For example, miR-223 was reported to be capable of inhibiting DENV-2 (strain TR1751) replication and its antiviral effect was likely associated with dampened expression of the microtubule-destabilizing protein, stathmin 1 (STMN1), a key microtubule regulatory protein that controls microtubule dynamics [77]. The exact mechanism of how STMN1 affected DENV-2 replication remains unclear. Nevertheless, insights from a previous study had demonstrated that an intact microtubule network involved in sequestration of STMN1 was essential for HCV to establish an infection. As such, it is highly probable that STMN1 is playing a similar role in establishing controlled microtubule dynamics in the context of DENV infection [91]. Additionally, miR-133a was hypothesized to suppress DENV multiplication by direct targeting of the polypyrimidine tract-binding protein (PTB). PTB is a host cellular protein that has been proposed to act as RNA helicase by binding to the conserved sequence 1 and long stem-loop structures of the 3’UTR of DENV [92, 93].

Diosa-Toro (2017) identified a miRNA with moderate but significant antiviral property against DENV. In the study, miRNA-3614-5p was found to be upregulated in DENV-negative cells and its overexpression reduced DENV infectivity. This miRNA-3614-5p was found not only to reduce DENV but also West Nile virus infectivity. Adenosine deaminase acting on RNA 1 (ADAR1) was analyzed as one of the targets of miR-3614-5p and was demonstrated to stimulate DENV infectivity at early time points post-infection. Taken together, the study extended the knowledge of the contribution of human miRNAs in building the network of interactions between DENV and its human host cells [78].

In contrast, miRNAs could also modulate the host factors or immune response in a way that favour viral replication. Several miRNAs have been reported to positively modulate viral replications, such as miR-1 in Hepatitis B and miR-122 in HCV infections. Nevertheless, miR-146a was the only miRNA identified to date that had a proviral effect in response to dengue infection [79]. Interestingly, this miRNA was highly expressed in the primary and transformed monocytes (THP-1 cells), the major target cells for the initial attachment of DENV, implying a crucial role of miR-146a in establishing an infection. Further study into the molecular mechanism proposed that miR-146a enhanced DENV-2 (strain New Guinea C) replication by dampening the host IFN-β production which was mediated through targeting tumor necrosis factor receptor (TNFR)-associated factor 6 (TRAF6). Ideally, a regimen that could repress the level of miR-146a might reverse the virus-mediated immune response. Administration of the LNA-antagomir-146a was reported to significantly decrease Enterovirus A71 (EV-A71) replication in mice [94]. Pu et al. (2017) demonstrated overexpression of miR-146a significantly blocked DENV-2 (strain New Guinea C) by inducing autophagy and LNA-mediated inhibition of miR-146a was able to counteract the effects [95]. In light of this finding, miR-146a antagonist might be a suitable candidate to restore the host IFN activity against DENV. However, one important concern is the potential off-target effects associated with a skewed transcription of type I IFN. While type I IFN is essential in inhibiting viral replication through the induction of ISGs, inappropriate or excessive type I response could lead to detrimental effects. These damaging effects might be through direct tissue damage by apoptosis or immunopathology due to excessive inflammation [96]. This is relevant to dengue infection as excessive inflammation is the hallmark of fatal severe dengue. In another study, Kanokudom et al. (2017) found that the expression of miR-21 was significantly increased upon DENV-2 (strain 16,681) infection which promoted DENV-2 replication [80]. This result was similar to the effect of miR-146a [79]. A significant reduction in DENV-2 production was observed upon treatment with an anti-miR-21 (AMO-21) prior to DENV infection in HepG2 cells, indicating that miR-21 played a crucial role in DENV-2 replication. However, the mechanism of how miR-21 induced DENV replication is still unclear and miR-21 might directly target NS1 protein sequence of DENV-2 genome [97].

miRNAs identified to date are mostly associated with DENV replication, either via direct targeting of the viral genome or through modulation of the host mediators which are essential for DENV multiplication. Although some studies have shown that the DENV viral load had a positive correlation with dengue severity [98–100], other studies have proposed that the dengue viral load was not significantly associated with DHF and dengue fatality [75, 82, 84]. Severity in dengue disease is strongly associated with a systemic inflammatory response syndrome, commonly known as the “cytokine storm”. It is a phenomenon which occurs when there is an imbalance between the pro-inflammatory and anti-inflammatory mediators. However, whether the immune responses are skewed towards either type 1 or type 2 is paradoxical [101]. Pro-inflammatory cytokines such as TNF-α are believed to activate the endothelial cells and increased vascular permeability. Sera from dengue patients were found to contain apoptosis-inducing factors which might also play a role in vascular damage [102]. Activation of the T lymphocytes is the downstream responses of DENV infection. Considering the diachronic course of the T cell responses and clinical manifestation of severe dengue, the cytokine profiles obtained from patients suggested that the key players of the massive release of pro-inflammatory mediators were the monocytes, macrophages and other non-T-lymphocytes. However, T cell activation might contribute to the cytokine storm in a subgroup of patients [101].

In light of this, a few studies were devoted to understand the effects of miRNAs on cytokine dysregulation during dengue infection. Chen et al. (2014) [81] demonstrated that miR-150 that was highly expressed in DHF patients was able to negatively regulate SOCS1, a suppressor of cytokine signaling protein whose aberrant expression could lead to cytokine dysregulation. Interestingly, they showed a reciprocal correlation between SOCS1 expression and dengue severity. In light of the demonstrated association between SOCS1 and miR-150, it is tempting to speculate that by controlling the overexpression of miR-150 in dengue patients, the expression of SOCS1 could be restored and the dysregulation in cytokine expression was rescued [81]. Indeed, SOCS1 was shown to be involved in immune regulation during VSV infection [103]. They reported that the suppression of SOCS1 by miR-155 enhanced type I IFN signaling, thereby suppressing viral replication. This strengthens the notion that by modulating specific miRNAs, non-optimal immune responses in patients could be potentially controlled.

Efforts were also directed to examine the association between differentially expressed miRNAs and highly elevated cytokines following DENV-2 (strain New Guinea C) infection [104]. Qi et al. (2013) showed that downregulation of miR-106b, miR-20a and miR-30b during DENV-2 infection might relieve inhibition of target genes, resulting in the increased levels of pro-inflammatory cytokines. The decrease of miR-106b due to its downregulation could have led to derepression of CCL5 and hence increased secretion of this cytokine. This study offers new insights into possible therapeutic approaches to attenuate the robust immune response of the host to DENV infection [104].

Overall, the host miRNAs can act as either proviral or antiviral via several mechanisms (Table 1). Depending on the abundance of miRNAs in the host, the impacts of proviral or antiviral effects can vary. For instance, the high expression of the proviral miR-146a significantly facilitated viral replication by targeting TRAF6 and reducing IFN-β production [79]. Dengue severity was associated with rapid initiation of innate host response and this innate antiviral mechanism mediated by IFN is potentially an important pathway of host defense in limiting viral replication. Besides, different miRNAs produced during DENV infection are associated with the severity of the disease through cytokine expressions. It has been suggested that cytokine storm leads to increased plasma leakage seen in DHF and DSS. Several miRNAs have been reported to be regulating cytokine production. For example, increased miR-150 was significantly associated with DHF concomitant to the suppression of SOCS1 expression, a negative regulator of several cytokines [81]. miR-let-7e has been shown to potentially target the mRNAs of IL-6 and CCL3 while miR-106b, miR-451, and miR-4279 are targeting mRNAs of CCL5 and CXCL1. Specific differentially expressed miRNAs appear to cause de-repression of cytokine expressions, while additional miRNAs target epigenetic modulators of cytokine expression [104]. However, further studies should be carried to determine the molecular mechanisms that induce the cytokine storm following DENV infection as there is a crucial gap in understanding the regulation of dengue disease outcomes.

## Limitations and delivery of miRNA

Present understanding of the role of miRNAs in the pathogenesis of dengue is restricted by insufficient characterization of the miRNA-associated molecular pathways in dengue infection, replication and host immunity [105]. The detection of miRNA targets within a living organism and in host defense processes with many layers of molecular and cellular elements remains a major challenge. One of the challenges in miRNA based therapies is the off-target effects since a single miRNA can target multiple mRNAs. Pathophysiological effects observed by altering their roles might be associated with changes in the levels of various target mRNAs [105]. Delivery of miRNAs is one of the key issues that might limit miRNA mimics and inhibitors being delivered precisely to the infected and inflamed tissues. This limitation would need to be overcome as it is crucial not to cause any disturbance to the normal functioning of the surrounding tissues. Ongoing development of modified miRNAs and suitable carriers are required to overcome these problems [106]. However, to date, the delivery of most miRNA has only been focused on the intended pathological tissues and organs of interest. The side effects and toxicities have commonly been overlooked but this has to be addressed as safety is a critical concern when investigating a new drug [107].

In addition, miRNA might also activate an innate immune response which is nonspecific and is associated with the structure, length, chemical modification, cellular localization and concentration instead of the base sequence. Immune activation triggered by miRNAs could have a negative effect on the health of the recipient. Thus, a few novel strategies have been developed to reduce the miRNA induced immune responses such as through the use of modified nucleotides (LNAs). Although it has not progressed to clinical trials, in vivo animal experimentation on the use of LNA-based miR-146a inhibitor against EV-A71 showed that it could prevent death in mice by restarting the production of type I IFN. At the early stage of viral infection, prophylactic administration of LNA antagomiR-146a was effective. This demonstrates the potential in vivo application of LNA for treatment against EV-A71 infection. Since miR-146a is also present in DENV, in vivo experimentation in the murine model is much needed to evaluate the therapeutic potential of miR-146a.

## Conclusion

Molecular understanding of host-virus interaction is essential in the development of more efficient antiviral strategies. Considering the intrinsic regulatory role of miRNAs, the hypothesis that miRNAs are playing an important role in regulating viral pathogenesis has been the basis of miRNA-related studies on viral infections. Indeed, this hypothesis has been well supported in dengue infection and the roles of miRNAs are discussed in this review. Understanding and elucidating the roles of miRNAs in fundamental processes associated with DENV infection are necessary to fully characterize their potentials in disease diagnosis and prognosis as well as disease treatment. The recent study of miRNAs might provide the tools for identifying common key genes and pathways that could be activated, repressed or enhanced in members of Flaviviruses. This would facilitate the identification of common targets for therapeutic interventions. Despite the continued efforts of the research community to seek antiviral agents against dengue infections, there is currently no effective antiviral treatment against all four DENV serotypes. As such, miRNAs that have been shown to affect DENV replication and pathogenesis do present opportunities as the targets for miRNA-based antiviral therapeutics for DENV infection. Nevertheless, the existing miRNA research on DENV remains at the target identification stage. In light of the advanced stage of development of Miravirsen, an oligonucleotide which has been shown to inhibit the function of miR-122a in HCV, this miRNA-based therapeutic has been proven to be a promising strategy to delve into, especially at a stage where an effective anti-DENV therapy is still lacking. miR-146a could be taken up as low-hanging fruit for research in the near future as it is the only human miRNA induced by different flaviviruses such as DENV and Japanese encephalitis virus (JEV), as well as in several other viral infection models including enterovirus, VSV and hepatitis B virus [108–110]. Mammalian cells that were infected with DENV-2, EV-A71, JEV or VSV produced miR-146a, implying a potential role of targeting this miRNA in viral infections [79, 94, 109, 111]. Thus, inhibition of miR-146a could reverse the virus-mediated immune response and by blocking the miRNA, it could serve as the most straightforward and easy approach to develop new antivirals. These findings indicate that miR-146a might serve as a promising therapeutic target for dengue.

To date, there is no in vivo studies in animals or clinical trials being initiated for any of the miRNAs that have been observed to have antiviral activity against dengue infection. Further studies are needed to verify whether the miRNAs known to have antiviral effects against DENV serotypes in vitro will exhibit anti-DENV effects in vivo and whether they are able to protect mice from lethal challenges with DENV serotypes 1–4. Without known targets in vivo in an animal model, the development of miRNAs as promising targets of intervention against dengue is still exploratory. Moreover, research should focus on special delivery systems that target specific tissues to reduce therapeutic doses and unwanted side effects. The selection of the lead miRNA to develop into an antiviral therapeutic will have to depend on both in vitro and in vivo performance in animal models. Thus, efficient in vivo delivery is critical for their successful use in an animal model. The use of nanocarriers and new non-invasive imaging techniques should be developed to monitor the in vivo distributions of the therapeutic nanocarriers that might help to optimize the treatment efficacy. The miRNAs have to maintain high stability in tissues by having good pharmacological properties and displaying no toxicity at the concentrations being administered. Besides these desirable attributes, the miRNA should not display off-target effects. Ongoing development of modified miRNAs and suitable nanocarriers are required to overcome these problems. The development of non-viral systems for the delivery of miRNA has undergone extensive expansion in recent years, providing solutions for the challenges hampering miRNA therapeutic success. The most advanced approach for systemic delivery was via lipid nanoparticles as a carrier system which is currently ongoing in Phase I-III human clinical trials. Nevertheless, the delivery of miRNA to organs other than the liver has been challenging and the toxicity of the nanocarriers is of concern. Thus, preclinical evaluations of miRNA administration using animal models is necessary to determine the efficiency of miRNA delivery to other organs besides the liver, as well as examining the potential safety.

## Data Availability

Not applicable.

## Abbreviations

ADAR1: Adenosine deaminase acting on RNA 1
ADE: Antibody-dependent enhancement
Ago: Argonaute
BACH1: Basic leucine zipper transcription factor 1
DENV: Dengue virus
DHF: Dengue haemorrhagic fever
DSS: Dengue shock syndrome
HCV: Hepatitis C virus
IFN: Interferon
ISG: Interferon stimulated genes
JEV: Japanese encephalitis virus
LNA: Locked nucleic acid
MDA5: Melanoma differentiation-associated protein 5
miRNA: MicroRNA
mRNA: Messenger RNA
NS: Non-structural protein
PRRs: Pattern recognition receptors
PTB: polypyrimidine tract-binding protein
RIG-1: Retinoic acid-inducible gene I protein
SOCS1: Suppressor of cytokine signaling
STAT1: Signal transducer and activator of transcription 1
STMN1: Stathmin 1
TLR: Toll-like receptor
TRAF6: Tumor necrosis factor receptor (TNFR)-associated factor 6
UTR: Untranslated regions
VSV: Vesicular stomatitis virus
